# Retrograde Coronary Venous Infusion as a Delivery Strategy in Regenerative Cardiac Therapy: an Overview of Preclinical and Clinical Data

**DOI:** 10.1007/s12265-018-9785-1

**Published:** 2018-02-01

**Authors:** Wouter A. Gathier, Dirk Jan van Ginkel, Mira van der Naald, Frebus J. van Slochteren, Pieter A. Doevendans, Steven A. J. Chamuleau

**Affiliations:** 1 Department of Cardiology, Division Heart and Lungs, University Medical Center Utrecht, Heidelberglaan 100, 3584 CX Utrecht, The Netherlands; 2 Regenerative Medicine Center Utrecht, Uppsalalaan 8, 3584 CT Utrecht, The Netherlands

**Keywords:** Cell therapy, Myocardial infarction, Heart failure, Retrograde coronary venous infusion

## Abstract

An important aspect of cell therapy in the field of cardiac disease is safe and effective delivery of cells. Commonly used delivery strategies such as intramyocardial injection and intracoronary infusion both present with advantages and disadvantages. Therefore, alternative delivery routes are explored, such as retrograde coronary venous infusion (RCVI). Our aim is to evaluate safety and efficiency of RCVI by providing a complete overview of preclinical and clinical studies applying RCVI in a broad range of disease types and experimental models. Available data on technical and safety aspects of RCVI are incomplete and insufficient. Improvement of cardiac function is seen after cell delivery via RCVI. However, cell retention in the heart after RCVI appears inferior compared to intracoronary infusion and intramyocardial injection. Adequately powered confirmatory studies on retention rates and safety are needed to proceed with RCVI in the future.

**Electronic supplementary material:**

The online version of this article (10.1007/s12265-018-9785-1) contains supplementary material, which is available to authorized users.

## Introduction

Cell therapy has proven to be safe and feasible for treatment of cardiac disease. Yet, the clinical relevance of cell therapy is uncertain. Recent meta-analyses show a marginal (2–5%) increase of cardiac function measured by left ventricular ejection fraction (LVEF) [1, 2]. Taking into account the dynamic nature and the high perfusion characteristics of the cardiac tissue [3], an important aspect of cell therapy is the location and mode of delivery. Two commonly used administration techniques are intramyocardial (IM) injection and intracoronary (IC) infusion [1, 2]. IM injection has the benefit of targeted delivery of cells in a target region, e.g., the border zone of the infarct [4], but this procedure is time-consuming, suffers from rapid wash-out of cells via venous drainage after injection [3], and needs specific systems in the catheterization laboratory. IC infusion is quick and easy to perform but the coronary system is often diseased in the target population, leading to inaccessibility of coronary arteries. Manipulation inside the coronary artery can potentially induce embolisms leading to decreased coronary blood flow [5–7]. Therefore, alternative delivery routes are explored. The coronary venous system is easily accessible and typically free of atherosclerotic disease. Retrograde coronary venous infusion (RCVI) is considered to be a good alternative to IM and IC administration. RCVI is performed by placing a balloon-catheter in the coronary sinus (CS) or into one of the coronary veins. In order to maximize the therapeutic potential, the balloon is kept inflated temporarily to prevent the loss of infused cells due to antegrade venous flow and to allow the cells to disseminate in the heart. For optimal effect, this occlusion is often prolonged for a certain period after cell infusion. Our aim is to provide a complete overview of preclinical and clinical studies applying RCVI as a cell delivery strategy and focus on safety aspects and efficiency measures.

## Methods

### Search Strategy and Eligibility

**Fig. 1:**
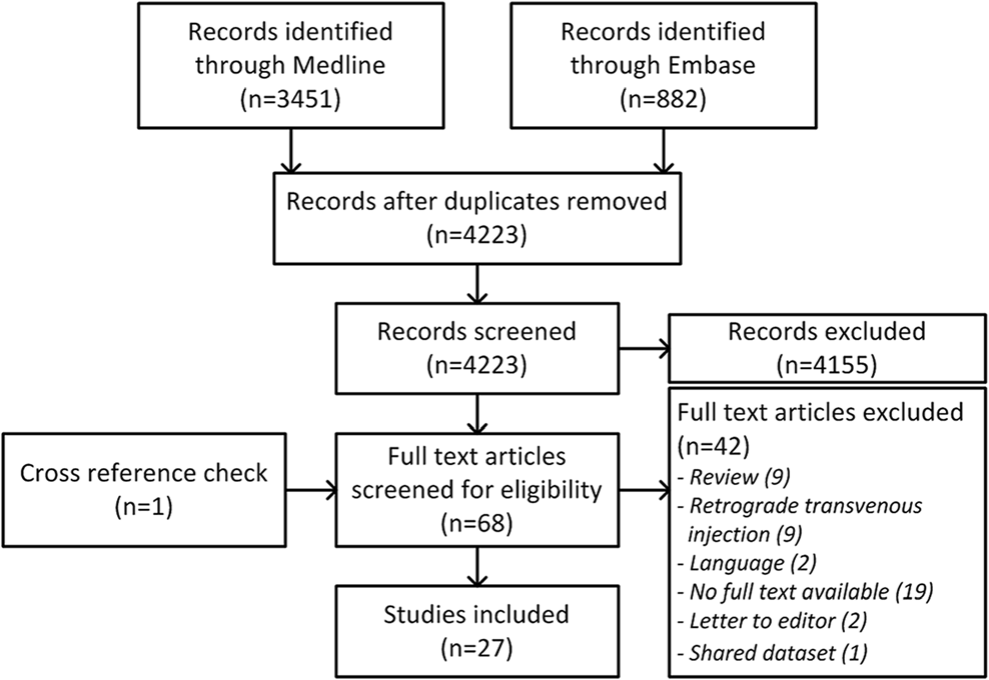
Flowchart of the systematic search

The full search strategy is available as Online Resource 1. In brief, we have performed a search using the PubMed and Embase databases on May 15, 2017. Trials were eligible for inclusion if they met the following criteria: (1) original (preclinical or clinical) study, (2) full text available in English, (3) covering cell therapy, (4) investigating safety or efficacy of retrograde CS/venous administration. An additional cross-reference screening was performed of included articles. The flowchart of the search is presented in Fig. 1.

## Results

### Search Results

The entire search yielded a total of 4333 (3451 Medline and 882 Embase) hits, of which 110 reports were removed after duplicate screening. Another 4155 reports were excluded after title/abstract screening because they did not fulfill the inclusion criteria. The remaining 68 articles were screened on the availability of full text, leading to another 42 exclusions. One article was excluded due to a shared dataset [8]. The cross-reference screening led to one additional inclusion that did not come up in the original search due to the absence of one part of the search string in the title and abstract [9]. The total number of articles included in this review is 27 (Fig. 1). All articles were published between 2003 and 2016.

### Preclinical and Clinical Experience

Retrograde coronary venous infusion has been performed in a number of different studies. In total, 21 preclinical studies are included in this review; 8 rat studies [10–17], 3 dog studies [18–20] and 10 pig studies [9, 21–29]. Patients were treated in 6 studies [30–35].

#### Preclinical Experience

Treatment was given in acute (acute myocardial infarction (AMI)) [9, 13–15, 19, 20, 22–25, 29] and chronic setting (chronic myocardial infarction (CMI)) [10–12, 17, 21, 26–29] and in chronic heart failure (CHF) [18]. One study treated healthy subjects (*n* = 1) [16]. Cell products administered included skeletal myoblasts (*n* = 6) [10, 12, 15, 16, 21, 26], bone marrow mononuclear cells (*n* = 2) [11, 29], peripheral blood mononuclear cells (*n* = 2) [22, 24], adipose-derived stem cells (*n* = 3) [18, 23, 24], mesenchymal stem cells (*n* = 6) [13, 14, 19, 20, 25, 27], embryonic endothelial progenitor cell (*n* = 1) [9], autologous unfractionated bone marrow (*n* = 1) [28], and cardiac explant-derived c-Kit+ cells (*n* = 1) [17]. One study administered both adipose-derived stem cells and peripheral blood mononuclear cells [24].

#### Clinical Experience

In the clinical setting, treatment was given in AMI [31], CHF [30, 32], and chronic refractory angina (CRA) [33–35]. Infused cell products included bone marrow mononuclear cells (*n* = 3) [30, 31, 33], umbilical cord subepithelial cells (*n* = 1) [32], and autologous unfractionated bone marrow (*n* = 2) [34, 35].

Table 1 shows study characteristics on disease model, recipients, and used cell type and number. In summary, there is broad experience with RCVI across species, disease models, and used cells.

**Table 1:** Practical aspects of RCVI regarding disease type, location of infusion, and infused cell type and number

	Study	Species	Number of subjects	Model	Administration	Cell type	Number of cells
Small animals	Di Lascio [10]	Rat	66	CMI	RCV	SMB	2 × 10^6 /100 g
	Fukushima [11]	Rat	35	CMI	RCV	BMMNC	10^7
	Fukushima [12]	Rat	85	CMI	RCV	SMB	5 × 10^6
	Huang [13]	Rat	90	AMI	RCV	MSC	10^6
	Huang [14]	Rat	38	AMI	RCV	MSC	10^6
	Suzuki [15]	Rat	62	AMI	RCV	SMB	10^6
	Suzuki [16]	Rat	20	NP	RCV	SMB	10^6
	Zakharova [17]	Rat	32	CMI	RCV	CEDC	10^6
Large animals	Pogue [18]	Dog	15	CHF	RCV	ASC	10^7
	Sun [19]	Dog	28	AMI	RCV	MSC	10^7
	Wang [20]	Dog	18	AMI	RCV	MSC	10^8
	Formigli [21]	Pig	15	CMI	RCV	SMB	8 × 10^7
	Hagikura [22]	Pig	15	AMI	RCV	PBMNC	5 × 10^6
	Hong [23]	Pig	7	AMI	RCV	ASC	10^7
	Hou [24]	Pig	5	AMI	RCV	PBMNC/ASC	10^7
	Kupatt [9]	Pig	ns	AMI	RCV	EEPC	5 × 10^6
	Lu [25]	Pig	36	AMI	RCV	MSC	10^8
	Prifti [26]	Pig	15	CMI	RCV	SMB	Ns
	Sato [27]	Pig	13	CMI	RCV	MSC	10^7
	Vicario [28]	Pig	16	CMI	RCS	AUBM	Ns
	Yokoyama [29]	Pig	21	AMI & CMI	RCV	BMMNC	3.2 ± 1.2 × 10^9
Clinical trials	Patel [30]	Human	46	CHF	RCS	BMMNC	3.7 × 10^9
	Silva [31]	Human	9	AMI	RCV	BMMNC	10^8
	Tuma [33]	Human	14	CRA	RCS	BMMNC	8.2 × 10^8
	Tuma [32]	Human	18	CHF	RCS	UCSEC	1×, 2×, 4 × 10^8
	Vicario [34]	Human	14	CRA	RCS	AUBM	0,04 or 0,08 × 10^8/kg
	Vicario [35]	Human	15	CRA	RCS	AUBM	>0,04 × 10^8/kg

*CMI* chronic myocardial infarction (administration of cells > 1 week post MI), *AMI* acute myocardial infarction (administration of cells up to 7 days post MI), *CHF* chronic heart failure, *NP* no pathology, *CRA* chronic refractory angina, *MI* myocardial infarction, *SMB* skeletal myoblasts, *BMMNC* bone marrow mononuclear cells, *PBMNC* peripheral blood mononuclear cells, *ASC* adipose-derived stem cells, *MSC* mesenchymal stem cells, *EEPC* embryonic endothelial progenitor cells, *UCSEC* umbilical cord subepithelial cells, *AUBM* autologous unfractionated bone marrow, *CEDC* cardiac explant-derived c-Kit+ cells, *RCV* retrograde coronary venous infusion, *RCS* retrograde coronary sinus infusion, *ns* not specified

### Practical Aspects of RCVI

There is a high degree of heterogeneity in the way that RCVI is performed. Important differences between models are (1) the infusion duration, (2) the volume of infused cell suspension, (3) the time that the CS or coronary vein is occluded to prevent cells from draining directly into the right atrium, (4) the number of cells infused, and (5) the location of infusion (Tables 1 and 2).

**Table 2:** Heterogeneity regarding practical aspects of RCI both within and between species

Study type	Infusion duration (min)	Infused volume (ml)	Occlusion time (min)
Rat studies (*n* = 8)	1.0 [0.5–1.0] (*n* = 3)	1.0 [0.5–1.0] (*n* = 8)	5.0 [1.0–5.0] (*n* = 8)
Dog studies (*n* = 3)	No data (*n* = 0)	10.0 [10.0–20.0] (*n* = 3)	Insufficient data (*n* = 2)
Pig studies (*n* = 10)	10.0 [0.25–40.0] (*n* = 9)	15.0 [10.0–25.0] (*n* = 10)	10.0 [5.0–20.0] (*n* = 7)
Human studies (*n* = 6)	5.0 [4.0–6.0] (*n* = 6)	60.0 [40.25–120.0] (*n* = 6)	15.0 [11.0–17.0] (*n* = 5)
Overall (*n* = 27)	5.0 [0.88–11.25 (*n* = 18)	10 ml [1.0–40.0] (*n* = 27)	10.0 [5.0–12.75] (*n* = 22)

Data are presented as median with interquartile ranges calculated using IBM SPSS statistics 21

*min* minute(s), *ml* milliliter(s), *n* number of studies that statistics are based on

#### Preclinical Experience

Cells are predominantly infused via the coronary veins in preclinical trials. The infused cell number ranged from approximately 1 × 10^6 to 3 × 10^9. Infusion duration, infused cell volume, and the time that the CS or coronary vein was occluded differed both within and between animal species (Tables 1 and 2).

#### Clinical Experience

In clinical trials, cells were mainly infused via the CS. The amount of cells infused was generally higher, ranging from approximately 1 × 10^8 to 4 × 10^9 cells. Notable differences between preclinical and clinical trials are that infused cell volumes were many times greater in clinical trials compared to preclinical trials and that the CS or coronary vein was occluded longer in clinical trials (Tables 1 and 2).

We found a striking reporting difference regarding practical aspects of RCVI, with roughly 20% of studies not adequately describing procedural characteristics. This hampers the possibility to repeat certain experiments if desired.

### Safety Issues

Here, safety is described as occurrence of arrhythmias related to RCVI, elevation of heart enzymes, cardiac tamponade, presence of pericardial fluid, microvascular obstruction (MVO), damage to the CS, and mortality. It should be noted that some studies did not report safety aspects due to the purpose and setup of these studies.

#### Safety Aspects Other than Mortality

##### Preclinical Experience

Thirteen preclinical studies reported safety aspects of RCVI. One study only described that RCVI is safe without providing data on safety [29]. Seven studies only reported absence of arrhythmias without providing in-depth data [10, 14–16, 22, 26, 28]. Five articles provided more in-depth data on safety aspects of RCVI [11, 12, 18, 20, 23]. These five studies will be discussed in more detail below.

In two studies, IM injection was associated with an increased chance of both spontaneous ventricular tachycardias and ventricular premature contractions after cell administration compared to RCVI, suggesting that RCVI is safer in these experimental models [11, 12]. Another study closely monitored dogs for occurrence of arrhythmias and reported transient atrial fibrillation during CS catheterization in 6 out of 15 dogs and a pre-existent ventricular arrhythmia in one dog [18]. In another dog study, no occurrence of arrhythmias or cardiac tamponade associated with RCVI was seen [20]. RCVI did not lead to MVO after cell administration in one pig study [23].

##### Clinical Experience

All six clinical studies reported safety aspects of RCVI. Two studies only reported absence of arrhythmias without providing in-depth data [34, 35]. The other four studies provided more in-depth information on safety. In one clinical trial, absence of arrhythmias associated with RCVI was reported, but a rise in cardiac enzymes was seen in some patients after RCVI [30]. Rise in cardiac enzymes after RCVI was also reported in some patients in another clinical trial [31]. In a population of patients with heart failure, a transient increase in Troponin-I levels was seen in all patients that resolved within 24 h after catheterization. No arrhythmias were seen in this patient population and there was no evidence of damage to the CS after infusion [32]. No occurrence of arrhythmias, no rise in cardiac enzymes, and no pericardial effusion after retrograde delivery of cells was seen in patients with chronic refractory angina [33].

#### Mortality

##### Preclinical Experience

Mortality rates were reported in 16 articles, with no RCVI-related deaths occurring in 11 of these 16 studies. The available mortality data are difficult to interpret because it is likely that other factors besides RCVI, such as surgical procedure, have had influence on mortality rates. Loss of subjects that could possibly be attributed to RCVI was seen in 5 studies, described below.

A loss of 11/66 rats (16.7%) after RCVI was seen in one study. This loss could be attributed to the fact that a thoracotomy was performed to access the coronary vein and might not be related to the RCVI procedure itself. Since all animals received cells through RCVI, there is no control group for mortality [10]. A comparison was made between mortality rates after IM injection and RCVI in two rat studies. Mortality rates were comparable between IM injection and RCVI with the first study showing mortality rates of 2/34 rats (5.9%) after IM injection and 2/35 rats (5.7%) after RCVI [11]. Similar results were seen in the second study with a mortality of 4/48 rats (8.3%) in the IM injection group compared to 4/49 rats (8.2%) in the RCVI group [12]. Surgical stress and bleeding were suggested to be the cause of mortality. A common complication with RCVI in small animals is sustained bleeding from the catheter insertion site because the catheter has to be inserted into the fragile left cardiac vein via the left superior vena cava or CS. A comparison was made between conventional RCVI and a modified method of RCVI to see if bleeding could be limited in small animals. Conventional RCVI was described as delivery of cells by direct insertion of a catheter in the left cardiac vein via the CS. Modified RCVI was described as cardiac vein catheterization via the left internal jugular vein. A mortality of 3/7 rats (42.9%) was seen in the group that received cells via conventional RCVI versus 0/20 rats (0%) in the group with modified RCVI [14]. One small animal study reported a loss of 18/62 rats (29%) within 24 h after RCVI, which the authors linked to development of acute heart failure rather than the RCVI [15].

##### Clinical Experience

In all six clinical trials, mortality rates were reported but mortality related to RCVI did not occur.

In conclusion, there seems to be no relation between the way RCVI is performed and the occurrence of adverse events, arrhythmias, and mortality. Especially large animal studies and clinical trials do not report mortality or arrhythmias related to RCVI. Although RCVI is reported to be safe in the majority of studies presented here, safety data on RCVI are underreported with the majority of studies providing no or insufficient safety data to conclude that RCVI is a safe method for cell delivery in the heart. Safety and mortality data are provided in Table 3.

**Table 3:** Safety and mortality data

	Study	Species	Reported safety aspects	Mortality related to retrograde infusion procedure
Small animals	Di Lascio [10]	Rat	No arrhythmias, described as safe	16,7% (11/66) probably related to thoracotomy)
	Fukushima [11]	Rat	More VPC and VT in IM group vs RCVI group, described as safe	RCVI: 5.7% (2/35) vs IM: 5.9% (2/34)
	Fukushima [12]	Rat	More VPC and VT in IM group vs RCVI group, described as safe	RCVI: 8.2% (4/49) vs IM: 8.3% (4/48)
	Huang [13]	Rat	ns	ns
	Huang [14]	Rat	No arrhythmias	conventional technique: 42.9% (3/7) modified technique: 0
	Suzuki [15]	Rat	No arrhythmias, described as safe	29% (18/62) within 24 h, probably due to acute heart failure
	Suzuki [16]	Rat	No arrhythmias	0%
	Zakharova [17]	Rat	ns	0%
Large animals	Pogue [18]	Dog	Transient AF during procedure in 6/15 dogs, described as safe	0%
	Sun [19]	Dog	ns	0%
	Wang [20]	Dog	No arrhythmias, no cardiac tamponade, described as safe	0%
	Formigli [21]	Pig	ns	0%
	Hagikura [22]	Pig	No arrhythmias, described as safe	0%
	Hong [23]	Pig	No MVO, described as safe	0%
	Hou [24]	Pig	ns	0%
	Kupatt [9]	Pig	ns	ns
	Lu [25]	Pig	ns	ns
	Prifti [26]	Pig	No arrhythmias, described as safe	0%
	Sato [27]	Pig	ns	0%
	Vicario [28]	Pig	No arrhythmias	ns
	Yokoyama [29]	Pig	Described as safe	ns
Clinical trials	Patel [30]	Human	Rise in cardiac enzymes in some patients, no arrhythmias associated with RCVI, described as safe	0%
	Silva [31]	Human	Rise in cardiac enzymes in some patients	0%
	Tuma [33]	Human	No rise in cardiac enzymes, no arrhythmias, no pericardial effusion, described as safe	0%
	Tuma [32]	Human	No arrhythmias, rise in cardiac enzymes in all patients, no evidence of CS leak or damage, described as safe	0%
	Vicario [34]	Human	No arrhythmias, described as safe	0%
	Vicario [35]	Human	No arrhythmias, described as safe	0%

*VPC* ventricular premature contraction, *VT* ventricular tachycardia, *IM* intramyocardial injection, *RCVI* retrograde coronary venous infusion, *ns* not specified, *AF* atrial fibrillation, *MVO* microvascular obstruction, *CS* coronary sinus

### Efficiency Measures

#### Retention Rate

##### Preclinical Experience

The therapeutic benefit of cell therapy is in part based on the retention of cells in the heart. In total, eight preclinical studies provide data on the percentage of administered cells that retain in the heart after RCVI (Table 4). Different methods are used to determine cardiac retention of cells. One method is the use of real-time polymerase chain reaction for the Y-chromosome-specific Sry gene to detect the amount of transplanted male cells in female subjects. Other methods include administration of β-galactosidase-expressing cells, or to label cells radioactively with ^111^Indium or Tc^99m^-hexamethylpropylenamineoxime for quantitative analysis using scintigraphy. The retention rates show a high degree of heterogeneity that can partially be explained by differences in animal model, disease model, cell type, infusion time point, follow-up time point, and quantification technique. Most studies report a retention ≤ 10% and two studies report a remarkably higher retention of respectively 31.4 ± 4.8 and 29.8 ± 6.9% [15, 16]. The latter studies applied an indirect measurement of retention by using β-galactosidase-expressing cells, and comparing the level of β-galactosidase activity to the standard curve. One study used a method to optimize retention (magnetic targeting) that resulted in an increase of retention from approximately 2% after routine RCVI to 8.5% with magnetic targeting [13]. It should be noted that the three large animal experiments [9, 23, 24] consist of very small sample sizes. RCVI appeared to be either inferior to [23, 24] or equal to [11, 12] IM injection or IC infusion regarding cell retention. Retention rates in Table 4 are presented as the percentage of total administered cells that is retained in the heart. In one study [23], retention of cells in the heart was reported as a percentage of cells retained in five major thoracoabdominal organs. We converted the data to a percentage of total administered cells that are retained in the heart in order to achieve comparability between studies. If retention of cells was measured at multiple time points, we reported retention at the first time point, because retention decreased in time in the majority of these studies. A decrease was not seen in three studies [15, 16, 23]. This can be explained by the fact that two of these studies used expression of β-galactosidase as a measure of cardiac cell retention [15, 16]. Increased expression of β-galactosidase over time was attributed to proliferation of administered cells. The third article [23] presented the retention of cells in the heart as a percentage of the total retention in five major organs. A possible explanation for the increase in retention at a later time point could be that the decrease in the number of cells in the heart was relatively less than the decrease in the number of cells in the five major organs, making this decrease in the heart look like an increase [23].

**Table 4:** Retention of cells in the heart

	Study	Species	#	Retention method	Retention time point	Application method				
						RCVI retention	IC retention	IM retention	Peripheral IV retention	Sign comparison
Small animals	Fukushima [11]	Rat	35	Sry	3 days	1.84 ± 0.27%	–	1.45 ± 0.27%	–	ns
	Fukushima [12]	Rat	85	Sry	3 days	10 ± 5%	–	14 ± 5%	–	ns
	Huang [13]	Rat	90	Sry	24 h	2%/8.5%^a^	–	–	–	*P* < 0.001^d^
	Suzuki [16]	Rat	20	β-galactosidase	10 min	31.4 ± 4.8%	–	–	–	na
	Suzuki [15]	Rat	62	β-galactosidase	10 min	29.8 ± 6.9%	–	–	–	na
Large animals	Hong [23]	Pig	7	Radiolabel	1 h	±8%^c^	±25%^c^	–	–	*P* = 0.037
	Hou [24]	Pig	5	Radiolabel	1 h	3.2 ± 1%	2.6 ± 0.3%	11.3 ± 3%	–	Not sign^b^
	Kupatt [9]	Pig	6	Radiolabel	1 h	2.7%	–	–	0.5%	ns
Clinical trials	Silva [31]	Human	9	Radiolabel	4 h	4.62%	16.14%	–	–	*P* = 0.01

In case retention was not measured as % of total administered dose (e.g., as a % of uptake in major organs), we calculated the retention % of total administered dose. This was the case in one study [23]

*Sry* polymerase chain reaction for the Y-chromosome-specific Sry gene, *β-galactosidase* presence of β-galactosidase-expressing cells, *radiolabel* retention measured by scintigraphy after radiolabeled cell infusion, *RCVI* retrograde coronary sinus/venous infusion, *IC* intracoronary infusion, *IM* intramyocardial injection, *IV* intravenous, *ns* not specified, *na* not applicable, *#* number of subjects

^a^2% in case of normal delivery, 8.5% in case of magnetic targeting

^b^Comparison between RCVI infusion and IM retention

^c^Corrected for total injected dose

^d^Normal delivery versus magnetic targeting

##### Clinical Experience

Retention of cells in the heart was determined in one clinical trial, showing inferiority of RCVI versus IC infusion [31]. Cells labeled with Tc^99m^-hexamethylpropylenamineoxime were used to assess retention in the heart. Just like the three pig studies, sample size was small and retention rates were comparable [9, 23, 24].

#### Functional Outcomes

The goal of cardiac reparative therapy is improvement of cardiac function or decrease of disease characteristics such as angina complaints in order to improve quality of life and decrease mortality. Here, we focused on the effect of cell administration on (1) LVEF (AMI, CMI, CHF), (2) improvement on the Canadian Cardiovascular Society scale (CSS) (CRA), and (3) myocardial perfusion (CRA).

##### Preclinical Experience

Most of the preclinical studies that reported changes in LVEF (12/15) showed a significant increase in LVEF versus baseline and/or controls. Three studies only showed improvement of LVEF when cells were combined with growth factors [22] or no effects on LVEF at all [18, 19].

##### Clinical Experience

Three out of four clinical studies reported significant improvement of LVEF. The study that did not show improvement of LVEF after RCVI compared IC infusion with RCVI and reported that patients receiving cells through IC infusion did show improvement in LVEF [31]. The difference in cell retention between IC infusion and RCVI in these patients might be the explanation for this difference in functional outcome. Two other studies show comparable retention rates between IM injection and RCVI and both groups show comparable functional gains [11, 12]. In case of CRA, changes in CCS scale and improvement in myocardial perfusion were reported [33–35].

In the majority of cases, cells administered with RCVI are able to effectuate improvement of cardiac function in a range of different experimental models. An overview of functional outcomes is presented in Online Resource 2.

## Discussion

Cell delivery strategies should meet two important demands. First and foremost, the technique should be safe. Second, it should be effective in delivering cells to the heart. In this paper, we provided an overview of RCVI.

There is a high degree of heterogeneity regarding technical aspects of RCVI both between and within species. Furthermore, roughly 20% of studies do not adequately describe procedural characteristics, which hampers the possibility to repeat these experiments technically.

The main finding is that relevant data regarding technique and safety are poorly reported. For instance, 30% of included studies do not report on safety aspects of RCVI at all, while 33% only report absence of arrhythmias without mentioning other safety parameters. Only a limited number of studies provide more in-depth safety information regarding RCVI. The six clinical trials included in this overview report cardiac enzyme rise as the only safety issue associated with RCVI and show no arrhythmias associated with RCVI, no development of pericardial fluid, and no sustained damage to the CS after RCVI. It is understandable that the first priority of research focused on cell therapy lies with validating the effectiveness of cell therapy in itself. From this perspective, it is logical that some studies do not report on safety of delivery because this was not the purpose of the study. Nevertheless, due to the poor reporting of safety aspects, we cannot make an accurate assessment of the safety profile of RCVI.

However, retrograde accessing of the coronary venous system has been performed for a long time in the field of cardiac surgery in a great number of patients. With retrograde cardioplegia (RC), the myocardium is retrogradely perfused during cardiac surgery to induce cardiac arrest and protect the myocardium. With RC, a balloon-catheter is used to occlude the opening of the CS, in a way comparable to RCVI. RC is reported to be safe, with injury to the CS occurring in 0.06 to 0.6% of patients [36, 37], resulting in formation of hematoma on the atrioventricular groove, perforation of the CS wall, pericardial effusion, or laceration of the right ventricle or CS [37–40]. These data would suggest that the technical part of RCVI, namely the insertion of a balloon-tipped catheter in the CS followed by infusion of fluid, should be safe.

Cells delivered through RCVI are able to improve cardiac function and alleviate angina symptoms. However, in terms of cell retention, the data suggest that RCVI is a limitedly effective delivery strategy for cell therapy. In fact, IC infusion and IM injection show either higher or equal retention rates. It is likely that inferior retention rates decrease the efficacy of RCVI.

Due to the limited number of studies included in this review, we cannot conclude that RCVI is favorable in certain disease types or that certain cell types performed better than others in the included studies.

In conclusion, the available data on technical and safety aspects of RCVI are insufficient and incomplete. Furthermore, retention data show inferior results compared to IC infusion and IM injection. We conclude that at present, there are not enough arguments to proceed with this technique in the clinical arena. Well-designed confirmatory studies on retention rates and safety data are required to proceed with RCVI in the future.

## Electronic Supplementary Material

Online Resource 1 (PDF 420 kb)

Online Resource 2 (PDF 473 kb)

## Abbreviations

AMI: Acute myocardial infarction
CCS: Canadian Cardiovascular Society scale
CHF: Congestive heart failure
CMI: Chronic myocardial infarction
CRA: Chronic refractory angina
CS: Coronary sinus
IC: Intracoronary
IM: Intramyocardial
LVEF: Left ventricular ejection fraction
MVO: Microvascular obstruction
RC: Retrograde cardioplegia
RCVI: Retrograde coronary venous infusion
