# Supplementary material for: Retrograde Coronary Venous Infusion as a Delivery Strategy in Regenerative Cardiac Therapy: an Overview of Preclinical and Clinical Data
Source: J Cardiovasc Transl Res. 2018 Feb 1;11(3):173–81. doi: 10.1007/s12265-018-9785-1 (PMC5973989; doi:10.1007/s12265-018-9785-1)
Supplement: Supplementary file 1 — (PDF 420 kb) [file 12265_2018_9785_MOESM1_ESM.pdf]

Online Resource 1: Search Identification

Retrograde coronary venous infusion as a delivery strategy in regenerative cardiac therapy: an overview of preclinical and clinical data

Wouter A. Gathier, M.D.[1], Dirk Jan van Ginkel, BSc[1], Mira van der Naald, M.D.[1], Frebus J. van Slochteren, MSc, PhD[1], Pieter A. Doevendans, M.D., PhD[1,2], Steven A.J. Chamuleau, M.D., PhD[1,2#]

[1]Department of Cardiology, University Medical Center Utrecht, Heidelberglaan 100, 3584 CX, Utrecht, the Netherlands
[2]Regenerative Medicine Center Utrecht, Uppsalalaan 8, 3584 CT, Utrecht, The Netherlands

[#]Email address for correspondence:
S.A.J.Chamuleau@umcutrecht.nl

PubMed and EMBASE libraries were searched for relevant articles on May 15th 2017. Duplicates were removed using RefWorks. Any duplicates that were not properly removed with RefWorks were excluded by hand. After duplicate removal, title/abstract screening was performed followed by full text screening. One article was later added that was found through cross-reference check. Only full text original papers published in English were included in this review. Studies that used transvenous injection were not included in this review. Two researchers were involved in the search and screening phase: W.A. Gathier and D.J. van Ginkel. The search string is presented in the appendix of this article. Figure 1 shows the flowchart depicting the search outcome.

PubMed:
Retrograde intravenous[Title/Abstract] OR Retrograde venous[Title/Abstract] OR Coronary sinus[Title/Abstract] OR Coronary vein[Title/Abstract] OR Coronary veins[Title/Abstract] OR Coronary venous[Title/Abstract] OR Coronary vessel[Title/Abstract] OR Coronary vessels[Title/Abstract] OR Retrograde intracoronary[Title/Abstract]

AND

Retroinfusion[Title/Abstract] OR Retro-infusion[Title/Abstract] OR Infusion[Title/Abstract] OR Infusions[Title/Abstract] OR Infused[Title/Abstract] OR Infusing[Title/Abstract] OR Delivery[Title/Abstract] OR Delivered[Title/Abstract] OR Delivering[Title/Abstract] OR Deliver[Title/Abstract] OR Administer[Title/Abstract] OR Administered[Title/Abstract] OR Administering[Title/Abstract] OR Administration[Title/Abstract] OR Route[Title/Abstract] OR Routes[Title/Abstract]

Embase:
'Retrograde intravenous':ti,ab OR 'Retrograde venous':ti,ab OR 'coronary sinus':ti,ab OR 'coronary vein':ti,ab OR 'coronary veins':ti,ab OR 'coronary venous':ti,ab OR 'coronary vessel':ti,ab OR 'coronary vessels':ti,ab OR 'retrograde intracoronary':ti,ab AND [embase]/lim NOT [medline]/lim

AND

'retroinfusion':ti,ab OR 'retroinfusions':ti,ab OR 'retro-infusion':ti,ab OR 'retro-infusions':ti,ab OR 'infusion':ti,ab OR 'infusions':ti,ab OR 'infusing':ti,ab OR 'infused':ti,ab OR 'deliver':ti,ab OR 'delivered':ti,ab OR 'delivery':ti,ab OR 'delivering':ti,ab OR 'administer':ti,ab OR 'administered':ti,ab OR 'administering':ti,ab OR 'administration':ti,ab OR 'route':ti,ab OR 'routes':ti,ab AND [embase]/lim NOT [medline]/lim

Two articles described the same dataset (Moreira et al. [1] and Silva et al.[2] of which only data from Silva et al. is included in this review to prevent double reporting of data. Two other articles describe the same patient dataset.[3,4] We decided to include both studies in this review because one of these articles presents one-year follow up data.[4] We ended up with a total of 28 articles, of which 27 were included in this review after exclusion of Moreira et al. All articles were published between 2003 and 2016.

1. Moreira Rde, C., Haddad, A. F., Silva, S. A., Souza, A. L., Tuche, F. A., Oliveira, M. A., et al. (2011). Intracoronary stem-cell injection after myocardial infarction: microcirculation sub-study. *Arq Bras Cardiol, 97*(5), 420-426.
2. Silva, S. A., Sousa, A. L., Haddad, A. F., Azevedo, J. C., Soares, V. E., Peixoto, C. M., et al. (2009). Autologous bone-marrow mononuclear cell transplantation after acute myocardial infarction: comparison of two delivery techniques. *Cell Transplant, 18*(3), 343-352, doi:10.3727/096368909788534951.
3. Vicario, J., Campos, C., Piva, J., Faccio, F., Gerardo, L., Becker, C., et al. (2004). Transcoronary sinus administration of autologous bone marrow in patients with chronic refractory stable angina Phase 1. *Cardiovasc Radiat Med, 5*(2), 71-76, doi:10.1016/j.carrad.2004.06.004.
4. Vicario, J., Campo, C., Piva, J., Faccio, F., Gerardo, L., Becker, C., et al. (2005). One-year follow-up of transcoronary sinus administration of autologous bone marrow in patients with chronic refractory angina. *Cardiovasc Revasc Med, 6*(3), 99-107, doi:10.1016/j.carrev.2005.08.002.
