# Supplementary material for: Retrograde Coronary Venous Infusion as a Delivery Strategy in Regenerative Cardiac Therapy: an Overview of Preclinical and Clinical Data
Source: J Cardiovasc Transl Res. 2018 Feb 1;11(3):173–81. doi: 10.1007/s12265-018-9785-1 (PMC5973989; doi:10.1007/s12265-018-9785-1)
Supplement: Supplementary file 2 — (PDF 473 kb) [file 12265_2018_9785_MOESM2_ESM.pdf]

Online Resource 2: Supplemental table 1: Functional outcomes after RCVI

Retrograde coronary venous infusion as a delivery strategy in regenerative cardiac therapy: an overview of preclinical and clinical data

Wouter A. Gathier, M.D.[1], Dirk Jan van Ginkel, BSc[1], Mira van der Naald, M.D.[1], Frebus J. van Slochteren, MSc, PhD[1], Pieter A. Doevendans, M.D., PhD[1,2], Steven A.J. Chamuleau, M.D., PhD[1,2#]

[1]Department of Cardiology, University Medical Center Utrecht, Heidelberglaan 100, 3584 CX, Utrecht, the Netherlands
[2]Regenerative Medicine Center Utrecht, Uppsalalaan 8, 3584 CT, Utrecht, The Netherlands

[#]Email address for correspondence:
S.A.J.Chamuleau@umcutrecht.nl

Study		Change in LVEF (%) myocardial perfusion or CCS	Follow up timepoint	Versus	P- value
Small animals	Di Lascio[1]	*LVEF: Echocardiopgraphy*<br>12 ± 11.2 % (cells)<br>16 ± 8.9 % (cells + RLX)	1 month	Baseline<br>Baseline	P=0.0016<br>P<0.0001
	Fukushima[2]	*LVEF: Echocardiopgraphy*<br>15.5 ± 1.7	7 days	Baseline	Described as significant
	Fukushima[3]	*LVEF: Echocardiopgraphy*<br>9.4 ± 0.9 %	7 days	Baseline	P<0.05
	Huang[4]	*LVEF: Echocardiopgraphy*<br>~5 %<br>~12 % (magnetic targeting)	3 weeks	PBS controls<br>PBS controls	P<0.05<br>P<0.05
	Suzuki[5]	*LVEF: Echocardiopgraphy*<br>10.7 ± 4.0 %<br>11.1 ± 3.7 %	4 weeks	PBS controls<br>Sham	P<0.05<br>P<0.05
	Zakharova[6]	*LVEF: Pressure volume derived*<br>11.4 ± 6.7 %	3 weeks	DMEM controls	P<0.05
Large animals	Formigli[7]	*LVEF: Echocardiography*<br>~17 % (cells)<br>~20 % (cells + RLX)	1 month	DMEM controls<br>DMEM controls	P<0.001<br>P<0.001
	Hagikura[8]	*LVEF: Pressure volume*<br>0.5 ± 3.8 % (cells)<br>9.7 ± 3.1 % (cells + VEGF)	4 weeks	Saline controls<br>Saline controls	Described as not significant<br>P<0.05
	Lu[9]	*LVEF: Echocardiopgraphy*<br>~5 % (cells + Adnull)<br>~11 % (cells + VEGF)<br>~27 % (cells + HGF)<br>*LVEF: SPECT*<br>~8 % (cells + Adnull)<br>~10 % (cells + VEGF)<br>~22 % (cells + HGF)	4 weeks	Baseline<br>Baseline<br>Baseline<br><br>Baseline<br>Baseline<br>Baseline	P<0.05<br>P<0.05<br>P<0.05<br><br>P<0.05<br>P<0.05<br>P<0.05
	Pogue[10]	*LVEF: Echocardiopgraphy*<br>-28.5 %	2 years[d]	Baseline	P<0.001
	Prifti[11]	*LVEF: Echocardiopgraphy*<br>12 ± 9.8 %<br><br>*LVEF: SPECT*<br>13 ± 8.6 %	1 month	Controls (no infusion procedure)<br><br><br>Controls (no infusion procedure)	P=0.001<br><br><br>P=0.001
	Sato[12]	*LVEF: Left ventriculography*<br>Preserved, no data<br>~12 %	4 weeks	Baseline<br>DMEM controls	No p-value<br>P<0.01
	Sun[13]	*LVEF: Echocardiopgraphy*<br>-0.1 ± 12.2 % (cells)<br>-3.1 ± 12.9 % (cells + bFGF)	40 days	Baseline<br>Baseline	Both described as not significant
	Wang[14]	*LVEF: Echocardiopgraphy*<br>~ 7 % (cells)<br>11 % (cells + bFGF)<br>14.9 ± 3.8 % (cells)<br>17.5 ± 3.3 % (cells + bFGF)	4 weeks	Baseline<br>Baseline<br>Saline controls<br>Saline controls	P=0.124<br>P<0.01<br>P<0.01<br>P<0.001
	Yokoyama[15]	*LVEF: Pressure volume derived*<br>~5 % (AMI)<br>~6 % (OMI)	4 weeks	Baseline<br>Baseline	P<0.05<br>P<0.05
Clinical trials	Patel[16]	*LVEF: Left ventriculography*<br>6.0 % (niCMP)<br>8.1 % (iCMP)<br>not specified (niCMP)<br>not specified (iCMP)	12 months	Baseline<br>Baseline<br>Controls, no infusion procedure<br>Controls, no infusion procedure	P=0.007<br>P=0.006<br>P=0.954<br>P=0.814
	Silva[17]	*LVEF: Radionuclide ventriculography*<br>0.4 ± 14.3 %	6 months	Baseline	P=0.88
	Tuma[18]	*LVEF: Echocardiopgraphy*<br>4.1 ± 7.4 % (100M cells)<br>11.3 ± 6.9 % (200M cells)<br>13 ± 6.5 % (400M cells)	12 months[a]	Baseline<br>Baseline<br>Baseline	P<0.05<br>P<0.05<br>P<0.05
	Tuma[19]	*CCS class*<br>1.4 ± 0.7<br>*Myocardial perfusion: SPECT*[c]<br>14.7 %<br>*LVEF: SPECT*<br>4.3 %	2 years[b]	Baseline<br><br>Baseline<br><br>Baseline	P<0.001<br><br>P=0.001<br><br>P=0.019

Supplemental table 2 Functional outcomes presented as improvement in LVEF and/or myocardial perfusion and/or CCS score

Baseline = timepoint before cell infusion, follow up timepoint = time after cell infusion, LVEF = left ventricular ejection fraction, RLX = relaxin, PBS = phosphate buffered saline, DMEM = Dulbecco's modified Eagle medium, VEGF = vascular endothelial growth factor, Adnull = null adenovirus, HGF = hepatocyte growth factor, SPECT = single-photon emission computed

tomography, bFGF = basic fibroblast growth factor, AMI = acute myocardial infarction, OMI = old myocardial infarction, niCMP = non-ischemic cardiomyopathy, iCMP = ischemic cardiomyopathy, M = million, CCS = Canadian Cardiovascular Society, ~: approximate, in case of figure only in source text

[a]: follow up also occurred at 1 and 4 months post cell infusion. The 100M cell group only showed significant increase in LVEF at 12 months post infusion

[b] follow up also occurred at 1 year

[c]: absolute reduction in ischemic myocardium

[d]: only 4/15 dogs were alive at this point. LVEF never significantly improved during 2 years

If changes in cardiac function had to be determined by calculating the difference between cardiac function at baseline and follow up or between cases and controls, the standard deviation of the change in cardiac function was calculated by taking the square root of the sum of the squares of the standard deviations belonging to cardiac function at baseline and at follow up, or cases and controls.

1. Di Lascio, G., Harmelin, G., Targetti, M., Nanni, C., Bianchi, G., Gasbarri, T., et al. (2012). Cellular retrograde cardiomyoplasty and relaxin therapy for postischemic myocardial repair in a rat model. *Tex Heart Inst J, 39*(4), 488-499.
2. Fukushima, S., Varela-Carver, A., Coppen, S. R., Yamahara, K., Felkin, L. E., Lee, J., et al. (2007). Direct intramyocardial but not intracoronary injection of bone marrow cells induces ventricular arrhythmias in a rat chronic ischemic heart failure model. *Circulation, 115*(17), 2254-2261, doi:10.1161/circulationaha.106.662577.
3. Fukushima, S., Coppen, S. R., Lee, J., Yamahara, K., Felkin, L. E., Terracciano, C. M., et al. (2008). Choice of cell-delivery route for skeletal myoblast transplantation for treating post-infarction chronic heart failure in rat. *PLoS One, 3*(8), e3071, doi:10.1371/journal.pone.0003071.
4. Huang, Z., Shen, Y., Sun, A., Huang, G., Zhu, H., Huang, B., et al. (2013). Magnetic targeting enhances retrograde cell retention in a rat model of myocardial infarction. *Stem Cell Res Ther, 4*(6), 149, doi:10.1186/scrt360.
5. Suzuki, K., Murtuza, B., Fukushima, S., Smolenski, R. T., Varela-Carver, A., Coppen, S. R., et al. (2004). Targeted cell delivery into infarcted rat hearts by retrograde intracoronary infusion: distribution, dynamics, and influence on cardiac function. *Circulation, 110*(11 Suppl 1), Ii225-230, doi:10.1161/01.CIR.0000138191.11580.e3.
6. Zakharova, L., Nural-Guvener, H., Feehery, L., Popovic, S., Nimlos, J., & Gaballa, M. A. (2014). Retrograde coronary vein infusion of cardiac explant-derived c-Kit+ cells improves function in ischemic heart failure. *J Heart Lung Transplant, 33*(6), 644-653, doi:10.1016/j.healun.2014.03.006.
7. Formigli, L., Perna, A. M., Meacci, E., Cinci, L., Margheri, M., Nistri, S., et al. (2007). Paracrine effects of transplanted myoblasts and relaxin on post-infarction heart remodelling. *J Cell Mol Med, 11*(5), 1087-1100, doi:10.1111/j.1582-4934.2007.00111.x.
8. Hagikura, K., Fukuda, N., Yokoyama, S., Yuxin, L., Kusumi, Y., Matsumoto, T., et al. (2010). Low invasive angiogenic therapy for myocardial infarction by retrograde transplantation of mononuclear cells expressing the VEGF gene. *Int J Cardiol, 142*(1), 56-64, doi:10.1016/j.ijcard.2008.12.108.
9. Lu, F., Zhao, X., Wu, J., Cui, Y., Mao, Y., Chen, K., et al. (2013). MSCs transfected with hepatocyte growth factor or vascular endothelial growth factor improve cardiac function in the infarcted porcine heart by increasing angiogenesis and reducing fibrosis. *Int J Cardiol, 167*(6), 2524-2532, doi:10.1016/j.ijcard.2012.06.052.
10. Pogue, B., Estrada, A. H., Sosa-Samper, I., Maisenbacher, H. W., Lamb, K. E., Mincey, B. D., et al. (2013). Stem-cell therapy for dilated cardiomyopathy: a pilot study evaluating retrograde coronary venous delivery. *J Small Anim Pract, 54*(7), 361-366, doi:10.1111/jsap.12098.
11. Prifti, E., Di Lascio, G., Harmelin, G., Bani, D., Briganti, V., Veshti, A., et al. (2016). Cellular cardiomyoplasty into infracted swine's hearts by retrograde infusion through the venous coronary sinus: An experimental study. *Cardiovasc Revasc Med, 17*(4), 262-271, doi:10.1016/j.carrev.2016.02.008.
12. Sato, T., Iso, Y., Uyama, T., Kawachi, K., Wakabayashi, K., Omori, Y., et al. (2011). Coronary vein infusion of multipotent stromal cells from bone marrow preserves cardiac function

in swine ischemic cardiomyopathy via enhanced neovascularization. *Lab Invest, 91*(4), 553-564, doi:10.1038/labinvest.2010.202.
13. Sun, Q. W., Zhen, L., Wang, Q., Sun, Y., Yang, J., Li, Y. J., et al. (2016). Assessment of Retrograde Coronary Venous Infusion of Mesenchymal Stem Cells Combined with Basic Fibroblast Growth Factor in Canine Myocardial Infarction Using Strain Values Derived from Speckle-Tracking Echocardiography. *Ultrasound Med Biol, 42*(1), 272-281, doi:10.1016/j.ultrasmedbio.2015.09.010.
14. Wang, X., Zhen, L., Miao, H., Sun, Q., Yang, Y., Que, B., et al. (2015). Concomitant Retrograde Coronary Venous Infusion of Basic Fibroblast Growth Factor Enhances Engraftment and Differentiation of Bone Marrow Mesenchymal Stem Cells for Cardiac Repair after Myocardial Infarction. *Theranostics, 5*(9), 995-1006, doi:10.7150/thno.11607.
15. Yokoyama, S., Fukuda, N., Li, Y., Hagikura, K., Takayama, T., Kunimoto, S., et al. (2006). A strategy of retrograde injection of bone marrow mononuclear cells into the myocardium for the treatment of ischemic heart disease. *J Mol Cell Cardiol, 40*(1), 24-34, doi:10.1016/j.yjmcc.2005.06.008.
16. Patel, A. N., Mittal, S., Turan, G., Winters, A. A., Henry, T. D., Ince, H., et al. (2015). REVIVE Trial: Retrograde Delivery of Autologous Bone Marrow in Patients With Heart Failure. *Stem Cells Transl Med, 4*(9), 1021-1027, doi:10.5966/sctm.2015-0070.
17. Silva, S. A., Sousa, A. L., Haddad, A. F., Azevedo, J. C., Soares, V. E., Peixoto, C. M., et al. (2009). Autologous bone-marrow mononuclear cell transplantation after acute myocardial infarction: comparison of two delivery techniques. *Cell Transplant, 18*(3), 343-352, doi:10.3727/096368909788534951.
18. Tuma, J., Carrasco, A., Castillo, J., Cruz, C., Carrillo, A., Ercilla, J., et al. (2016). RESCUE-HF Trial: Retrograde Delivery of Allogeneic Umbilical Cord Lining Subepithelial Cells in Patients With Heart Failure. *Cell Transplant, 25*(9), 1713-1721, doi:10.3727/096368915x690314.
19. Tuma, J., Fernandez-Vina, R., Carrasco, A., Castillo, J., Cruz, C., Carrillo, A., et al. (2011). Safety and feasibility of percutaneous retrograde coronary sinus delivery of autologous bone marrow mononuclear cell transplantation in patients with chronic refractory angina. *J Transl Med, 9*, 183, doi:10.1186/1479-5876-9-183.
